# Supplementary material for: Both Saccharomyces boulardii and Its Postbiotics Alleviate Dextran Sulfate Sodium-Induced Colitis in Mice, Association with Modulating Inflammation and Intestinal Microbiota
Source: Nutrients. 2023 Mar 20;15(6):1484. doi: 10.3390/nu15061484 (PMC10055518; doi:10.3390/nu15061484)
Supplement: Supplementary file 1 [file nutrients-15-01484-s001.zip › nutrients-2271140-supplementary.pdf]

Supplementary

Table S1. Disease activity index scoring criteria

Score	Body weight loss（%）	Fecal traits	Hematochezia
0	≤0	Normal stools	Normal
1	1-5	Soft stools	Small amounts of blood-streaked feces
2	6-10	Loose stools	A certain amount of bloody feces
3	11-15	Semi-loose stools	Conspicuous blood-wrapped feces
4	＞15	Watery stools	Visible rectal bleeding

The DAI calculating formulais as follows: DAI = 1/3 (Body weight loss score + Fecal traits score + Hematochezia score).

Table S2 Summary of major rodent studies investigating the protective effect of S. boulardii against dextran sulfate sodium (DSS)-induced ulcerative colitis.

Animals	Intervention method	Probiotic dose	Probiotic treatment duration	Time of probiotic application	Main observations	Reference
C57BL/6J mice (6 weeks old, male)-humanized mouse model	mixing *S. boulardii* in finely crushed chow and drying as food pellets	1 × 10^8 CFU/d	21 d	21 d before induction of colitis	Significantly ameliorated the colon damage and regulated inflammatory responses by modulating the cytokine profile.<br>*S. boulardii*-derived polysaccharides and polypeptides altered microbiome composition and short-chain fatty acid (SCFA) metabolism.	[18]
Female C57BL/6 mice (8 weeks old, 20-24 g)	oral gavage	1 × 10^5 or<br>1 × 10^7 CFU/d	21 d	21 d before induction of colitis	Reduced DAI, colon length shortening, and loss of histological structure<br>Protected the intestinal barrier, suppressed colonic inflammation, restored myeloperoxidase activity, mitigated colonic oxidative damage<br>Suppressed the nuclear translocation of NF-κB p65 subunit, promoted the nuclear translocation of nuclear factor erythroid 2-related factor 2 (Nrf2)	[19]
Male C57BL/6 mice (6–8 weeks old)	oral gavage	5 × 10^7 CFU/d	12 weeks	12 weeks after induction of colitis	Reduced tumor load and reduced TNF-*α* and IL-6 levels.<br>Significant changes in both fecal and mucosal microbiota.	[20]
Male C57BL/6J mice (6 weeks old)	oral gavage	0.46 mg/g/d	7 d	At the same time	Reduced the disease activity index and histological score<br>Exerted a protect effect on the expression of ZO-1<br>Decreased the level of TNF-*α* and IL-8<br>Preserved the tight junctions and ameliorated microvilli and inter-cellular space.<br>Higher percentage of Bacteroidetes and a lower percentage of Firmicutes<br>Increased the proportion of the S24-7 family	[21]
male BALB/c mice (6–8 weeks)	oral gavage	150 mg/kg/day	14 d	7 d before induction of colitis	Reduced DSS-induced weight loss, ameliorated the histological damage and protected the colon barrier.<br>Significantly inhibited the increase in hypoxia-inducible factors (HIFs) in the colon.	[22]
Male C57BL/6 J mice (7–9 weeks old; approximately 20 g)	oral gavage	5×10^9 CFU/d	26 d	14 d before induction of colitis	Ameliorated the colonic damage.<br>Modified the colonic expression of different inflammatory markers and the epithelial integrity proteins.<br>Induced changes in micro-RNAs expression.<br>Increased bacterial diversity, ameliorated dysbiosis.<br>Modulatied in the immune response.	[23]

female C57BL6 mice(8 weeks old)	oral gavage	6×10^8 CFU/d	5 d	At the same time	Attenuated weight-lossand histological damage.<br>Reduced the neo-vascularization associated with acute DSS colitis and accelerated mucosal recovery restoration of the lamina propria capillary network to a normal morphology.	[24]
male BALB/c mice (6–8 weeks)	oral gavage	1×10^7 CFU/d	7 d	At the same time	Decreased the severity of DSS-induced clinical scores and histological inflammation.<br>Decreased inflammation and C. albicans colonization.	[25]
